# Unique Molecular Alteration of Lobular Breast Cancer: Association with Pathological Classification, Tumor Biology and Behavior, and Clinical Management

**DOI:** 10.3390/cancers17030417

**Published:** 2025-01-27

**Authors:** Huina Zhang, Yan Peng

**Affiliations:** 1 Department of Pathology, University of Rochester Medical Center, Rochester, NY 14642, USA; 2 Department of Pathology and Simmons Comprehensive Cancer Center, University of Texas Southwestern Medical Center, Dallas, TX 75390, USA

**Keywords:** invasive lobular breast carcinoma, pathological classification, *CDH1* gene, E-cadherin, tumor biomarkers, clinical management

## Abstract

Invasive lobular carcinoma (ILC), accounting for up to 15% of diagnosed breast cancers, has garnered significant attention due to the loss of the epithelial cell–cell adhesion molecule E-cadherin. This loss contributes to its distinct biological, morphological, and clinical characteristics compared to non-lobular breast cancers. The use of immunohistochemistry (IHC) for E-cadherin and/or the associated cadherin–catenin complex, such as p120-catenin and beta-catenin, in morphologically equivocal cases, has been increasingly adopted in pathology practice. This approach has substantially improved diagnostic accuracy, interobserver reproducibility, and the identification of new morphologic variants of ILC. ILCs exhibit unique tumor biology, which presents considerable challenges in clinical management, especially in preoperative imaging evaluation, surgical management, and neoadjuvant treatment. Recent advances in translational and clinical research have enhanced our understanding of ILC and have spurred the development of new clinical trials specifically targeting these cancers. This review highlights recent progress in various aspects of ILC, including its unique molecular alteration, pathological classification and diagnostic approach, tumor biology and behavior, key clinical management challenges, and ongoing clinical trials, as well as the role of artificial intelligence in diagnosing ILC radiologically and pathologically. The goal of this review is to provide an updated understanding of the tumor biology, clinical manifestations, and molecular landscape of ILC and to help refine current tumor classification and diagnosis, subsequently improving management strategies and overall outcomes for lobular carcinoma patients.

## Simple Summary

Invasive lobular carcinoma (ILC) of the breast has been increasingly recognized as a biologically distinct subtype of breast cancer with unique molecular alteration and consequential implications on pathological diagnosis, tumor behavior, and clinical management strategies. In recent years, substantial efforts in translational and clinical research have advanced our understanding of the intricate biology underlying ILC. This article provides a comprehensive overview of the latest advancements in the field of ILC, focusing on its distinctive molecular alteration as well as refined pathological classification and diagnostic approaches. We also delve into the unique tumor biology and behavior of ILC, including tumor-infiltrating lymphocytes and prognostic multi-gene genomic profile, highlighting how these aspects significantly impact preoperative imaging evaluation, surgical management, and medical oncologic treatments. By integrating these insights, this review aims to contribute to the ongoing efforts to improve the diagnosis, treatment, and prognosis of patients with ILC.

## 1. Introduction

Invasive lobular carcinoma (ILC) is the second most common histologic subtype after invasive ductal carcinoma (IDC, also referred to as invasive breast cancer of no special type). It is the most common “special” subtype of breast cancer and represents up to 15% of diagnosed invasive breast cancers [1]. Morphologically, ILC is characterized by distinct features resulting from the loss of epithelial cell–cell adhesion molecule E-cadherin. Classic ILC is the most common subtype and demonstrates discohesive tumor cells arranged as single cells, single-file growth pattern, or sometimes in a concentric pattern around existing benign ducts and lobular units (targetoid pattern), with minimal stromal desmoplastic reaction. Beyond the classic type of ILC, approximately 10 histologic variants have been described based on tumor architecture or cytomorphology [1,2,3,4]. The loss of E-cadherin membrane expression, detected by immunohistochemistry (IHC), along with cytoplasmic localization of p120-catenin, is useful for the accurate pathologic classification of ILC [1,5]. However, the morphologic heterogeneity of ILC, inconsistent use of ancillary IHC, various IHC staining patterns, misinterpretation of IHC stain, as well as overlapping features with IDC lead to diagnostic challenges and inconsistent diagnosis [6,7,8,9,10]. Improving the reproducibility of ILC diagnosis and classification requires a deeper understanding of tumor histologic heterogeneity, phenotypes, molecular profiles, and the appropriate use and interpretation of ancillary studies.

In recent years, ILC has been increasingly recognized as a biologically distinct breast cancer with unique molecular alteration and consequential implications on diagnosis and clinical management. Compared to ductal carcinomas, lobular breast carcinomas manifest unique tumor biology and behaviors, such as a high likelihood of multicentric, multifocal, and bilateral growth in the breast, metastasis in unusual organ sites, late recurrence, higher percentage of hormone receptor positivity and poor response to neoadjuvant chemotherapy [11]. Despite distinct clinical, morphologic, and molecular characteristics, ILC has long been treated indistinguishably from IDC. With the advanced understanding of ILC, imaging monitoring strategies and clinical trials specifically targeting ILC are gaining an accelerating interest in recent years, which emphasizes the importance of accurate diagnosis of ILC. This review article highlights recent advances in ILC, including molecular alteration, pathological classification and diagnostic approach, tumor biology, phenotype and behavior, clinical management, as well as ongoing specific clinical trials.

## 2. Hallmark of Molecular Alteration of Invasive Lobular Carcinoma

It is well-known that the key molecular alteration of ILC is the loss of E-cadherin due to the inactivation of the *CDH1* gene [12,13,14,15,16,17,18,19,20,21]. *CDH1* gene is located at chromosome 16q22.1 and encodes E-cadherin, a 120 kD calcium-dependent cell–cell adhesion glycoprotein, which binds normal and polarized epithelial cells together at the lateral surface through adherent junctions [22,23]. E-cadherin glycoprotein is composed of five extracellular cadherin repeats, a transmembrane region, and a highly conserved cytoplasmic tail. The binding of alpha, beta, gamma, and p120-catenins to its cytoplasmic tail links E-cadherin to the actin cytoskeleton and sequesters catenin from participating in downstream signaling pathways [24,25]. The *CDH1* gene inactivation is seen in ~50–80% of ILCs through truncating mutation (frameshift indels, nonsense, or splicing mutations), non-truncating mutation (in-frame indels or missense mutations), possible *CDH1* promoter methylation, transcriptional repression, or post-translational modifications [13,14,15,16,17,18,19,20,21,26,27,28,29]. These mutations are scattered over the whole coding region, including pro-peptide/signal domain (~26.7%), extracellular domain (~58.9%), transmembrane domain (~3.3%), and intracytoplasmic domain (~11.1%) [21,27]. *CDH1* gene mutations in ILC commonly occur in combination with heterozygous loss of 16q (~89% of ILC cases), where *CDH1* is located (16q22.1), and induce the complete loss of E-cadherin protein [16]. In a small subset of ILCs without the absence of *CDH1* gene alteration, abnormalities in α-catenin (encoded by the *CTNNA1* gene) and other adhesion factors such as *CTNND1* and *AXIN2* may underlie the lobular phenotype [28,30]. The mutations in the *CDH1* gene and other adhesion molecules result in a lack of E-cadherin protein expression observed in ~90% of ILCs [20,31]. Loss of E-cadherin is usually accompanied by abnormal expression of other members of the cadherin–catenin complex, such as loss of membranous β-catenin expression and upregulation and re-localization of p120-catenin in cytoplasm [5,31,32,33].

## 3. Current Classification and Diagnosis of Invasive Lobular Carcinoma in Pathology Practice

The term of “lobular carcinoma in situ”, along with an invasive form (classic ILC), was first coiled by Foote and Stewart in 1941 [34]. Since the discovery of the loss of E-cadherin adhesion molecule in ILCs, it has been recognized that ILC has a variety of cytologic and architecture features, and several variants have been described in the 5th edition of the World Health Organization (WHO) classification of breast tumors based on growth pattern (i.e., solid, alveolar, tubulo-lobular) or cytomorphology (i.e., pleomorphic, histiocytoid/apocrine, signet ring) [1].

### 3.1. Classic Invasive Lobular Carcinoma

**Figure 1:**
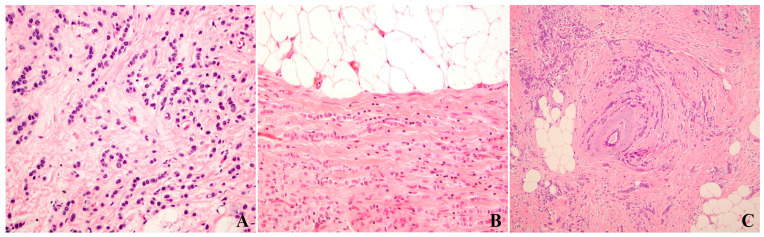
H&E sections of classic invasive lobular carcinoma highlight the single file rows of tumor cells with low nuclear grade ((**A**), 200×), partially fat-avoiding growth ((**B**), 200×), and occasional targetoid growth ((**C**), 100×).

Classic ILC is the most common form (~48–80%) of ILCs [29,35,36] and is composed of single cells or single-file rows of comparatively small (nuclear grade 1 or 2), discohesive tumor cells within the fibrous stroma. These tumor cells have monomorphic nuclei, scanty cytoplasm, occasional intracytoplasmic vacuoles, and rare mitotic figures. Occasionally, the tumor cells arrange in circular fashion around normal ducts and have “targetoid” appearance. Tumor-associated stromal desmoplasia is usually minimal or absent. A notable morphologic feature is that two tumor cells with round nuclei sandwich a tumor cell with two-sided concave nuclear compression and pronounced nuclear flexibility lying in between [37]. Focal pseudoductal formation, i.e., a few lumen-like spaces formed in a background of otherwise classical lobular carcinoma, can be rarely seen [38]. Tumor cells often infiltrate the adipose tissue with partially fat-avoiding growth of lobular tumor cells at the invasion front [37]. Figure 1 illustrates classic ILC, highlighting the single file rows of tumor cells with low nuclear grade and partially fat-avoiding or targetoid growth.

### 3.2. Histologic Variants of Invasive Lobular Carcinoma

ILC has a broad spectrum of morphologic patterns, and currently, at least 10 different histologic variants of ILC have been described based on cytomorphology or architecture [1,2,3,4]. By definition, these ILC variants demonstrate discohesive tumor cells with loss or aberrant E-cadherin membrane expression. Among them, pleomorphic and solid variants are the most common non-classic variants of ILC, accounting for approximately 18% of ILCs, and are associated with aggressive tumor behavior and a poor response to chemotherapy [36].

**Figure 2:**
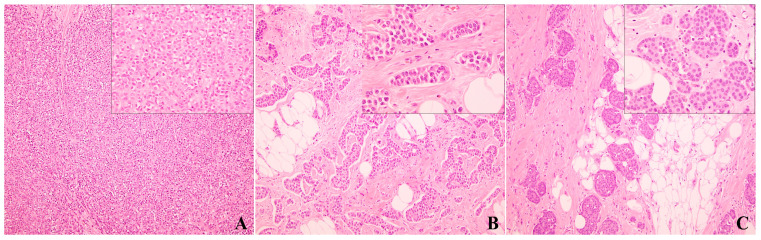
H&E sections of variants of invasive lobular carcinoma based on tumor architecture, including solid ((**A**), 100×), trabecular ((**B**), 100×), and alveolar ((**C**), 100×) variants (Insets: 400×).

Based on growth architecture, ILC has solid, trabecular and alveolar variants. The solid variant shows a solid growth pattern of discohesive cells with little or no intervening stroma (Figure 2A). The trabecular variant of ILC has also been described, which is composed of trabeculae of discohesive tumor cells two or three cells thick (Figure 2B). An alveolar variant refers to ILC with tumor-forming globular aggregates of 20 or more cells, separated by thin bands of collagenous fibrosis or normal stroma (Figure 2C), and can be easily mistaken for foci of atypical lobular hyperplasia or lobular carcinoma in situ. Figure 2 shows common variants of ILCs based on tumor architecture.

**Figure 3:**
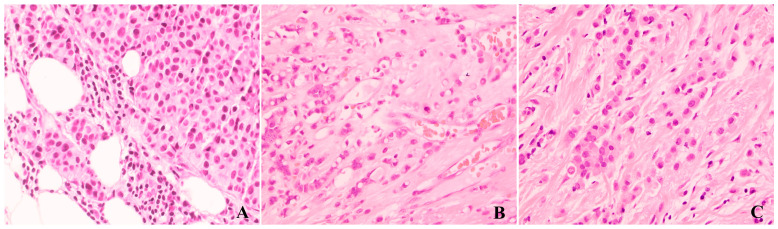
H&E sections of variants of invasive lobular carcinoma based on tumor cytology, including pleomorphic ((**A**), 400×), signet-ring cell ((**B**), 400×), and histiocytoid ((**C**), 400×) variants.

Based on cytomorphology, ILC has pleomorphic, apocrine, signet ring cell, and histiocytoid variants. Pleomorphic ILC is characterized by discohesive tumor cells with marked nuclear pleomorphism, defined as cell nuclei greater than 3–4 times the size of lymphocytes with variable presence of prominent nucleoli (Figure 3A). The apocrine variant refers to the discohesive tumor cells with abundant granular eosinophilic cytoplasm, eccentric round-to-oval nuclei, and prominent nucleoli. Signet ring cell variant refers to discohesive tumor cells with a single, large intracytoplasmic mucin-filled vacuole and a peripherally displaced and compressed crescent-shaped nucleus (Figure 3B). The signet-ring variant of ILC mimics gastric cancer or other metastatic tumors with signet-ring features to the breast. The histiocytoid variant was first described in a series of 13 breast cancers metastasized to the eyelid [39] and refers to ILC with relatively uniform discohesive tumor cells with single small eosinophilic nucleolus and ample granular, foamy, or ground glass cytoplasm (Figure 3C). The histiocytoid variant of ILC can be mistaken for histiocytes, granulation tissue, or granular cell tumors. Figure 3 shows common variants of ILCs based on tumor cytology.

Recently, two new variants of ILC were described, including ILC with extracellular mucin [2,40,41,42,43,44,45,46,47,48,49] and ILC with solid papillary growth pattern [3,4,50,51,52,53,54]. ILC with extracellular mucin was first reported in 2009 by Rosa et al. [2] and is a rare histologic variant of ILC. It is defined as invasive carcinoma with lobular morphology and the presence of extracellular mucin pools (range: 5–95%), which often presents with grade 3 nuclei and variant (signet-ring, solid, or apocrine) morphologic features [40]. Soong et al. studied 17 cases and revealed that ILC with extracellular mucin is a distinct variant of ILC with aggressive behavior and often has recurrent mutations/amplification, including *PIK3CA*, *POLQ*, *TP53*, *ERBB3*, *ERBB2*, *RUNX1*, *GATA3*, *FOXA1*, and *CCND1* [40]. ILC with a solid papillary growth pattern was originally described by Rakha et al. in 2016 [3] and has been rarely encountered. This ILC variant displays single or multiple well-circumscribed lesions comprised of a solid proliferation of lobular cells (confirmed by loss of E-cadherin membrane expression) with fibrovascular cores and tumor cells at the periphery usually arranged in a trabecular pattern akin to classic ILC. Due to the overlapping morphology, the diagnosis of ILC with solid papillary growth pattern on core biopsy has been misinterpreted as lobular carcinoma in situ, IDC with lobular features, and solid papillary carcinoma or encapsulated papillary carcinoma.

ILC with tubular elements was also recently described in a study of 13 cases, which shows non-cohesive invasive tumor cells admixed with cohesive tubular elements [55]. Interestingly, IHC showed E-cadherin was lost or markedly reduced in both non-cohesive tumor cells and admixed tubular elements in all cases, while beta-catenin expression was lost in non-cohesive tumor cells in all cases but was retained in tubular elements in the majority of cases (11/13). Further work-up demonstrated the presence of E-cadherin to P-cadherin switch in these cases, and P-cadherin was the molecular determinant of a mixed-appearing morphology in ILC with tubular elements [55]. Different from ILC with tubular element, tubulolobular breast carcinoma shows small tubules and cords of invasive tumor cells with both ductal and lobular features. Despite strong and uniform E-cadherin expression in tubulolobular carcinoma, it is listed as an ILC variant in the current WHO classification, and its precise classification remains controversial [1,56,57,58].

The definitions for ILC variants differ in the literature and these variants also overlap significantly between cytology and architecture. For example, different definitions for pleomorphic ILCs were proposed, including (1) all ILCs with nuclear pleomorphism greater than those seen in classic ILCs, which includes both nuclear grade 2 and 3 ILCs [59]; (2) ILCs with anaplastic nuclei, abundant cytoplasm and apocrine differentiation with large, lobulated, indented, and hyperchromatic nuclei [60]; (3) ILCs with nuclear grade 3 [61]; and (4) ILC with nuclei 4 × larger than a lymphocyte (nuclear diameter ≥ 18 µm), which is adopted in the current WHO classification [1,62]. These variants usually co-exist with classic ILC, and ILC with predominant variant growth is uncommon [63,64]. In addition, there is no specific cut-off for defining ILC variants, and some variants may not be considered distinct variants. Currently, the College of American Pathologists (CAP) reporting template does not require documenting the variant type of ILC in pathology reports.

### 3.3. Suboptimal Accuracy of Pathologic Classification of Invasive Lobular Carcinoma

The morphologic heterogeneity of ILC leads to inconsistent classification of ILC in pathology practice, and the diagnostic reproducibility of ILC based on morphology alone was reported to be fair to substantial, with the Cohen’s κ values ranging between 0.31 and 0.80 [6,7,8,9,10,65]. A 1999 study by Cserni. et al. reported that the kappa values for pure ILC and for the presence of an ILC component were 0.31 and 0.32 (fair agreement) among 10 pathologists and were 0.4 and 0.46 (moderate agreement) for pathologists experienced in breast pathology [6]. Studies from two recent large breast cancer clinical trials (MINDACT trial and WSG Plan B trial) revealed the concordance in ILC diagnosis between central pathology review and local laboratories reached only approximately 60% and 66% of cases [9,10]. Among common morphologic subtypes of ILC, the trabecular growth pattern was the most frequently associated with discordant classification [8].

### 3.4. Ancillary Immunohistochemistry in Diagnosing Invasive Lobular Carcinoma

**Figure 4:**
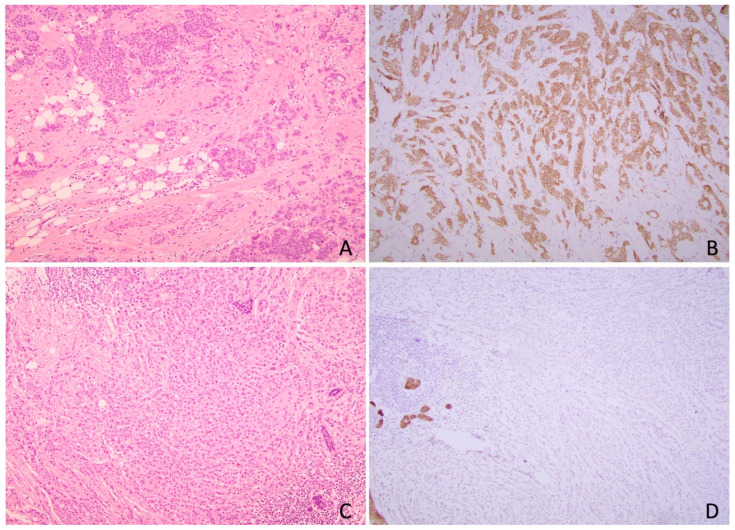
A case with one focus of invasive ductal carcinoma (**A**,**B**), and another focus of invasive lobular carcinoma (**C**,**D**). (**A**) H&E section of invasive ductal carcinoma (100×); (**B**) E-cadherin immunohistochemistry showing membrane staining (200×) in invasive tumor cells of ductal type; (**C**) H&E section of invasive lobular carcinoma (100×); (**D**) E-cadherin immunohistochemistry showing loss of membrane staining (200×) in invasive tumor cells of lobular type.

The hallmark of lobular lesions in the breast, including atypical lobular hyperplasia, lobular carcinoma in situ, and ILC is the loss of E-cadherin membrane expression [66,67]. The successful development of antibodies against E-cadherin for use in the formalin-fixed paraffin-embedded tissue in clinical practice significantly reduces the uncertainty in histologic typing and improves accurate pathologic classification and interobserver reproducibility of ILC [8,68,69]. Currently, E-cadherin IHC is commonly used in pathology practice to distinguish lobular lesions from ductal counterparts in morphologically indeterminate cases [70]. It is well established that the majority of ductal and lobular lesions would demonstrate expected differential patterns of staining for E-cadherin, with diffuse circumferential membranous staining in ductal lesions and complete loss of membranous staining in lesions with lobular phenotype (Figure 4). Although E-cadherin IHC is usually straightforward for interpretation to distinguish lobular from ductal lesions, there are up to 23.5% of ILC cases showing aberrant E-cadherin expression, including reduced (compared to staining intensity in normal lobules) or incomplete membranous (fragmented, focal, or beaded patterns) expression, dot-like perinuclear Golgi-type pattern, and diffuse cytoplasmic staining, likely due to the dysfunctional E-cadherin binding complex with underlying molecular alterations in *CDH1* [20,31,53,71]. The presence of these aberrant expressions of E-cadherin in ILCs could potentially lead to misinterpretation and subsequent histologic misclassification. Therefore, recognition of these aberrant E-cadherin expressions is important for proper tumor histologic classification.

**Figure 5:**
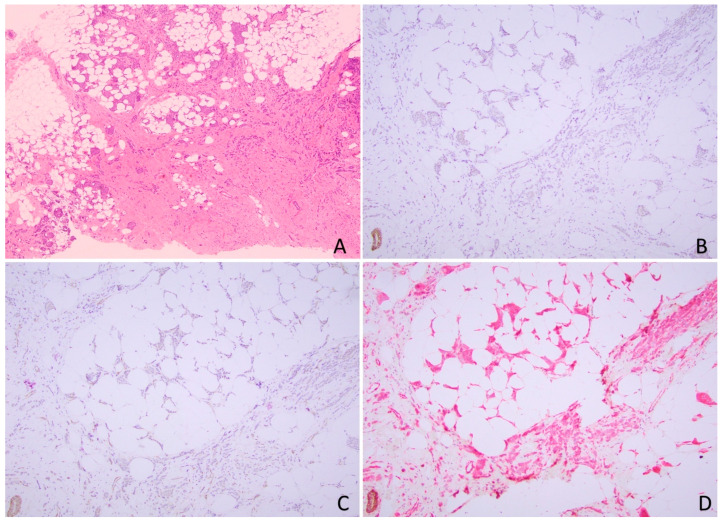
A case of invasive lobular carcinoma with equivocal morphology. (**A**) H&E section showing invasive tumor nests in fibrous and adipose tissue with focal linear growth. (100×); (**B**) E-cadherin immunohistochemistry showing loss of membrane staining in tumor cells (200×). (**C**) Beta-catenin immunohistochemistry shows loss of membrane staining in tumor cells (200×). (**D**) E-cadherin/p120-catenin cocktail immunohistochemistry showing cytoplasmic p120 expression (pink) with loss of E-cadherin membrane staining in tumor cells (200×).

In more challenging cases, especially in cases with discordant morphology and E-cadherin expression, additional IHCs for E-cadherin associated cadherin–catenin complex, including p120- catenin and beta-catenin have also been used as a supplement to E-cadherin IHC. The breast ductal lesions show complete membranous expression of beta-catenin and p120-catenin. In contrast, the cells of breast lobular lesions typically show absent, incomplete membranous or cytoplasmic expression of beta-catenin, with diffuse cytoplasmic and occasional nuclear (rather than membrane) expression of p120-catenin [5,71] (Figure 5). In general, the staining patterns of β-catenin and p120-catenin in ILC are in keeping with E-cadherin staining in most of the cases [69], and diffuse E-cadherin expression and abnormal β-catenin or p120-catenin staining occurs in a small subset of ILCs, representing the dysfunction of cadherin–catenin complex [21,69]. It needs to be noted that the loss of membranous staining of catenins, especially p120-catenin, can be seen in approximately 10% of IDC [72], and therefore staining for beta-catenin/p120-catenin alone without E-cadherin is not recommended [70].

Recently, Calhoun and Dabbs suggested that one potential approach to the use of IHC for the primary diagnosis of ILC would be to routinely stain for both E-cadherin and p120-catenin since p120-catenin is the only positive stain for tumors with lobular morphology [73]. In some pathology laboratories, including one of the author’s institutions (URMC), dual staining with an E-cadherin/p120-catenin cocktail is used to aid the diagnosis of ILC, and this approach may reduce the number of unstained slides used for diagnosis in tumors with limited sampling and preserve tissue for subsequent studies [73] (Figure 5D).

When examining the immunohistochemical stains for ductal vs. lobular phenotype, special attention should also be paid: (1) optimization of the E-cadherin/p120-catenin/beta-catenin immunostaining protocol to avoid both false-negative and false-positive staining; (2) the external and internal controls should be evaluated first to confirm appropriate staining, and performing IHC on same/different tissue block or different specimens should be considered if these controls do not demonstrate adequate staining; (3) when IHC studies are performed, thorough examination of the intensity and pattern of E-cadherin staining is important to distinguish aberrant staining from strong, complete membranous staining typically seen in ductal lesions [20]; (4) the tumor classification should not be based solely on the IHC result and correlation of E-cadherin staining pattern with tumor morphology is required to avoid the misinterpretation since benign ductal epithelium and intermingled myoepithelial cells display membranous E-cadherin expression [20,31,53]. In additional, reduced/loss of E-cadherin membranous expression occurs in a subset (5–10%) of high-grade IDC [74,75].

The expression pattern of E-cadherin is dependent on the antibody clone used. There is significant variability in the E-cadherin antibody clones and their associated staining procedures, including differences in concentration, antigen retrieval methods, and validation processes, which can lead to discrepancies in the staining results and their interpretation [70]. Studies have demonstrated in ILCs harboring *CDH1* gene mutations, the immunoreactivity for the anti-E-cadherin clone NCH38 was retained in 5% (8/156) of cases, and the immunoreactivity for the anti-E-cadherin clone 36 was retained in 23% (47/202) of cases [20,29]. Djerroudi et al. compared two commonly used clones for E-cadherin, clone 4A2C7 (directed against the intracellular domain) and clone NCH38 (directed against the extracellular domain), and found that the overall agreement between these two clones was 83.8% with a kappa value of 0.67 when using 3-tier categorization of E-cadherin IHC (null/focal (≤10%), heterogeneous (11–89%), and diffuse (≥90%)) [21]. A study by De Schepper et al. investigated five different antibody clones of E-cadherin (NCH-38, EP700Y, Clone 36, NCL-L-E-cad, and ECH-6), which demonstrated the overall agreement for complete membranous staining among these five antibodies ranged between 0.82 and 0.95 and the overall agreement for any degree of absent staining ranged between 0.75 and 0.95 [69].

By using clones 4A2C7 and NCH38, ILCs with *CDH1*-truncating mutations often show E-cadherin null/focal expression, while cases with *CDH1* non-truncating mutations are significantly correlated with diffuse E-cadherin expression [21]. The study of De Schepper et al. found there was no consistent association between the antibody clone and the location of the *CDH1* mutation (extracellular vs. cytoplasmic), although the results did find variant allele frequency and high probability of nonsense-mediated RNA decay of the *CDH1* gene were significantly associated with absent E-cadherin IHC [69]. Of note, in rare cases, the location of the *CDH1* gene mutation does influence the pattern of staining of the E-cadherin clones, regardless of the epitope used (extracytoplasmatic vs. intracytoplasmatic domain). For example, a nonsense mutation (c.2569C > T; p.Q857 *) located at the C-terminus of E-cadherin, which is beyond the binding site for beta-catenin and p120-catenin, results in the presence of the complete membrane staining of all clones of E-cadherin, beta-catenin, and p120-catenin [69].

Of note, according to the current WHO classification of tumors of the breast, the diagnosis of ILC is a morphological diagnosis and the essential morphologic criteria for the diagnosis of classic ILC are “dispersed or linear dyscohesive tumor cells with low-to-intermediate nuclear grade morphology and a low mitotic count” [1]. In addition, the presence of atypical lobular hyperplasia and/or lobular carcinoma in situ is considered desirable but not essential morphologic criteria by WHO [1]. The WHO does not require IHC for ILC diagnosis, and IHC for E-cadherin is listed as a desired criterion but not essential [1]. Results from a recent worldwide survey showed that 52% (75/143) of the participated institutions routinely perform IHC as an ancillary test to diagnose ILC, 45% (64/143) perform IHC in case of doubt, and only 3% (4/147) of the pathologists declared to never use IHC for the diagnosis of ILC [70]. The same study also reported that 50% (71/143) of the participants use E-cadherin only, 13% (19/143) use both E-cadherin and beta-catenin, 23% (33/143) use E-cadherin in combination with p120-catenin, and 11% (16/143) use all three antibodies [70]. The application of immunohistochemical stains against E-cadherin, in conjunction with p120 and/or beta-catenin, has significantly improved the interobserver agreement for the diagnosis of ILC [8,68,70].

### 3.5. Recommendations on Improving Diagnosis of Invasive Lobular Carcinoma in Pathology Practice

Since the diagnostic distinction between ductal and lobular lesions is important for clinical management, the recommendations from breast pathology experts to improve pathologic diagnosis of ILC [31,53,71] include: (1) E-cadherin should not be performed in in-situ or invasive carcinomas that display clear lobular morphology on hematoxylin and eosin-stained sections; (2) IHC for E-cadherin is very helpful in morphologically indeterminate (ductal vs. lobular) cases; (3) in cases in which IHC for E-cadherin fails to allow definitive categorization of a lesion as ductal or lobular, IHC for p120-catenin or beta-catenin can be performed to ascertain whether the E-cadherin is dysfunctional; (4) in cases that have equivocal histologic features and remain indeterminate on immunohistochemical analysis for E-cadherin, p120- and/or beta-catenin, the tumor should be diagnosed as in-situ or invasive carcinoma with ductal and lobular features. In rare cases where diagnostic uncertainty persists, *CDH1* mutational analysis may be of additional help in proper type categorization if needed.

Based on the characterization of five frequently used E-cadherin antibodies at the biochemical level on a collection of samples with different types of mutations in different exons, including the extracellular and intracellular domains of *CDH1* in a single laboratory, European Lobular Breast Cancer Consortium (ELBCC) recently recommended: (1) the mere presence of any type of *CDH1* mutation, or the evaluation of IHC without the consideration of tumor morphology, cannot be recommended as the “gold standard” for ILC diagnosis, which highlights the importance of the morphologic assessment as the first and leading step for the diagnosis; (2) based on overall adequate biochemical specificity, good quality of IHC staining in the internal control, and excellent diagnostic performance, a slight preference for E-cadherin clones NCH-38 and ECH-6 over other clones is recommended, although such preferences need to be confirmed in other laboratories and validated in clinical practice; (3) a complete membranous staining pattern for beta-catenin and p120-catenin in the tumor cells would be compatible with the diagnosis of ductal lesion, whereas an incomplete membranous, cytoplasmic or absent β-catenin staining pattern and cytoplasmic staining for p120 would be consistent with the diagnosis of lobular lesions. In cases where there is an admixture of (complete) membranous and non-membranous catenin staining, the diagnosis of a mixed IDC/ILC can be rendered in conjunction with tumor morphology [69].

## 4. Unique Tumor Biology of Invasive Lobular Carcinoma

### 4.1. Unique Clinical Presentation of Invasive Lobular Carcinoma

Women with ILCs are slightly older (approximately 3–4 years difference) than those with ductal carcinomas at the initial diagnosis (average age: 63 vs. 59 years) and tend to have larger tumor size with more frequent nodal metastasis [76,77,78]. ILCs tend to be multifocal [79,80]. Historically, it has been believed that ILCs occur bilaterally more often than ductal counterparts, with estimates ranging between 20% and 29% [81,82]. A recent meta-analysis of literature published between 2014 and 2021 showed among 599 patients with ILCs, bilateral diseases were found in 4.95% (range of 1.9–15%) of patients by histopathological examination and 10.2% by imaging [83]. ILCs tend to be initially indolent, but with slow progression and can recur even 10 years after initial diagnosis. In addition to metastasis in bone and liver, ILCs have a higher chance of metastatic dissemination in unusual organ sites such as the peritoneum, gastrointestinal tract, urinary tract, gynecologic tract, leptomeninges, skin, and orbit [84,85].

### 4.2. Phenotypic and Intrinsic Molecular Subtypes of Invasive Lobular Carcinoma

The majority (>90%) of ILCs, especially classic, alveolar, solid, and solid papillary variants, express hormone receptors (HR) and are negative for HER2 protein overexpression or *HER2* gene amplification, which are more likely to respond to the endocrine therapy [35,36,86,87,88,89,90]. Approximately 50% of classic ILCs are low histologic grade with luminal A molecular subtype and have favorable short-term outcomes. ILC with extracellular mucin is always ER-positive, and HER2 positivity is seen in a small subset (~10%) of cases [40]. ILC with tubular elements is also usually ER-positive (13/13) and HER2-negative (13/13) [55]. These uncommon variants of non-classic ILC are exceedingly rare, with limited clinical outcome data available in the literature.

Histiocytoid ILCs tend to have higher nuclear grade, and they are usually luminal B molecular subtype [91]. Pleomorphic and high-grade solid variants demonstrate high histologic grades and elevated Ki67 levels compared to classic ILC. They are associated with poor prognostic characteristics, higher risk of disease recurrence and death, as well as worse chemotherapy response [35,36]. Factors such as older patient age and negative HR status are significantly correlated with worse clinical outcomes in patients with pleomorphic ILCs [62]. Molecularly, compared to classic ILCs, pleomorphic ILCs tend to have an over-expression of HER2 and loss of ER expression, with *TP53*, *IRS2*, and *IGFR* mutations [92,93]. The solid variant often has more *ARID1A* mutations and *ESR1* gains, and the alveolar variant is characterized by gains in *CCND1* and *PAK1* genes [29].

HER2 protein overexpression/gene amplification is uncommon in ILCs, which occurs in ~5% of non-pleomorphic variants of ILCs and up to 30–40% of pleomorphic and apocrine variants [38,62,94,95,96,97,98,99]. HER2-amplified/overexpressed ILCs usually have higher tumor stage, more frequent positive nodal status, more distant metastases, and higher histological grade, and were more often HR negative when compared to HER2 negative ILCs [38,62,94,95,96,97,98,99]. *HER2* gene somatic mutations have been identified in a small subset of *HER2* gene non-amplified breast cancers and are rarely seen in *HER2* amplified cases [29,100,101]. Interestingly, *HER2* gene mutations are enriched in ILCs (2–15%), particularly in high-grade ILCs and metastatic ILCs, and are associated with early recurrence and increased resistance to endocrine therapy [98,101,102,103,104]. Triple-negative ILCs are rare (1–2%) and represent a group of tumors with high nuclear grade and heterogenous molecular subtypes, including 48% luminal A, 3% luminal B, 32% HER2 enriched, and 16% basal-like subtypes [105,106]. During disease metastasis, approximately 15%, 44%, and 5% of ER, PR, and HER2 experience receptor conversion between primary and paired metastatic lobular carcinomas, and metastatic lobular carcinomas tend to have reduced expression or loss of HR and increased HER2 expression [107].

Currently, it is also crucial to identify a subset of HER2 non-overexpressed/non-amplified breast cancers with HER2 IHC score of 1+ or 2+ and a negative in-situ hybridization result (HER2-low) to select suitable patients for treatment with the HER2-directed antibody-drug conjugate trastuzumab deruxtecan (T-DXd) [108]. Approximately 33–65% of ILCs were reported to have HER2-low status [109,110,111,112]. A study by Djerroudi et al. found that the clinicopathological features associated with HER2-low status in ILCs were older age, non-classic histological types, higher histologic grade, higher ER expression levels, and proliferation index compared to HER2-negative (IHC 0) group [112]. In a large cohort of 666 invasive breast cancers with lobular or mixed lobular and ductal histology, 65% of cases had HER2-low expression. Most clinicopathologic features did not significantly differ between HER2-low and HER2-negative (IHC 0) cases. However, when adjusting for variables including tumor size, number of positive nodes, ER/PR status, and treatment, patients with HER2-low status had worse disease-free survival than those with HER2-negative tumors (hazard ratio 2.0, 95% confidence interval 1.0–4.1, *p*  =  0.05) [111]. Interestingly, *ERBB3* mutation, although identified at a low frequency (7.1%), was the unique mutated gene exclusively associated with HER2-low ILCs [112].

In our opinion, tumor marker status plays a more critical role in determining tumor behavior and prognosis in the molecular era than traditional tumor grading. Advances in targeted therapies addressing tumorigenesis pathways in lobular cancer are important for improving survival outcomes in ILC patients. Further molecular characterization of ILCs, particularly pleomorphic variants, is essential for the development of targeted therapeutic strategies. Much work remains to be done to achieve this goal.

### 4.3. Prognostic Multi-Gene Genomic Profile of Invasive Lobular Carcinoma

Multi-gene genomic assays such as Oncotype DX, MammaPrint, PAM50, EndoPredict, and the Breast Cancer Index have been implemented to stratify the recurrence risk and assist the decision of chemotherapy treatment in ER+/HER2- early-stage breast cancer patients. The recurrence risks of breast cancer in most commercially available genomic assays were validated in predominantly ductal cancers, and their validity in ILC patients lacks substantial certainty [113]. Overall, the recurrence risk of most ILC cases tends to be in the low-to-intermediate range, and approximately 1.3–6.6% of the included patients had a high recurrence score on Oncotype DX assay [78,113,114,115]. The percentage of the high-risk group in ILC on MammaPrint assay was reported to be much higher (11–24%) than that by Oncotype DX assay, likely due to different study populations [113]. In addition, patients with ILCs tend to have a higher rate of discordance between genomic and clinical risks [116]. Abel et al. reported in 7399 HR+ /HER2- breast cancer patients (5902 IDC and 1497 ILC) with available MammaPrint assay results, patients with ILC had a significant chance of falling into a discordant risk category, compared to those with IDC (46.8% versus 37.1%, *p* < 0.001). Among ILC patients with discordant risk, clinical high/genomic low status accounted for ~76% of cases, especially in patients under the age of 50 years, which is likely due to the fact that there is a high rate of patients with “low-risk” tumors by MammaPrint presented at more advanced clinical stage [116].

### 4.4. Tumor-Infiltrating Lymphocytes in Invasive Lobular Carcinoma

Tumor-infiltrating lymphocytes (TILs) are an important component of the adaptive immune system and play significant roles in tumor progression and response to immune checkpoint blockade, including anti-PD-L1 immunotherapy. ILC has long been considered as immune quiescence. The first comprehensive retrospective analysis of a large multicentric ER+/HER2- breast cancer cohort by Desmedt et al. [117] reported that TIL levels in ILC were statistically significantly lower than those of IDC, with a median level of 5%. 15% of ILCs had greater than 10% TILs. High TIL levels were associated with poor prognostic characteristics, including young patient age, high Ki-67 proliferation index, and lymph node involvement. When compared to classic ILCs, the mixed non-classic ILCs and alveolar variants of ILCs had a significant increase and decrease in TILs, respectively [117].Tille et al. [64] investigated 459 ILCs and found 52% (239/459) of cases had no TILs, 40% (185/459) having ≤5% TILs, and 7.5% (35/459) having >5% TILs. Similar to the study of Desmedt et al., this study also showed TILs were associated with younger age, larger tumor size, lymph node involvement, poor Nottingham prognostic index, HER2 amplification, prominent nucleoli as well as poor overall survival and invasive disease-free survival. More importantly, TILs could identify a subset of ILC patients with poor overall survival independently of molecular subtype and lymph node metastases [64]. These studies suggest that TILs may represent a promising new morphologic biomarker in ILC.

A recent study on 66 pleomorphic ILCs revealed 64% of cases had ≥1% TILs, 36% had positive PD-L1 score of ≥1% by SP142 antibody, and 28% had a positive PD-L1 score of ≥1 by 22C3 antibody. However, the results from this study failed to demonstrate any correlation between TILs or PD-L1 expression and tumor size, tumor grade, nodal status, expression of ER, or amplification of HER2, as well as any difference in survival between the three molecular subtypes of pleomorphic ILC with respect to TILs and PD-L1 expression [118]. The presence of TILs and PD-L1 expression in a subset of ILCs suggests some ILC patients might benefit from immune checkpoint blockade. In a recently published phase II GELATO trial study (NCT03147040), among 23 patients with metastatic ILC treated with carboplatin and PD-L1 blockade atezolizumab, meaningful clinical benefit was achieved in 6 patients (26%), including 4 patients with a partial response (17%), and 2 with stable disease. Four of these six patients had triple-negative ILC [119]. Future studies are needed to better understand the tumor microenvironment in ILC, and identify biomarker which may help select patients for more targeted therapy.

## 5. Key Clinical Management Challenges Associated with Invasive Lobular Carcinoma

Although the current clinical management of ILC is essentially similar to that of its ductal counterpart, the unique tumor growth and biology of ILC significantly affect the clinical management, especially in the setting of preoperative imaging evaluation, surgery, and neoadjuvant treatment. The detailed clinical management of ILC has been comprehensively reviewed in two recently published articles [11,120]. Multidisciplinary therapeutic decision-making should incorporate the unique biology of ILC into clinical practice to personalize oncological breast cancer patient care further. In this review, we briefly summarized the key clinical management challenges associated with the unique tumor biology of ILC.

Conventional imaging modalities, including 2D mammography, digital breast tomosynthesis, and ultrasound, are less sensitive for detecting ILC [121]. The overall sensitivity of 2D mammography on the detection of ILC is ~34–83% in non-dense breast and of ~10% in women with dense breast tissue [122,123]. The sensitivity of ultrasound for detection of ILC is ~88–98% [124,125,126]. The radiologic detection of ILC has dramatically improved after the application of more sensitive imaging modalities such as magnetic resonance imaging (MRI) (sensitivity ~83–100%) and contrast-enhanced mammogram (CEM) (sensitivity 97–100%); however, there is insufficient evidence for widespread use of these new sensitive imaging modalities in clinical practice as part of breast cancer screening guidelines [121,127]. Additional imaging beyond mammography is warranted to screen for ILC in women with dense breast tissue and preoperative breast MRI is recommended to evaluate extent of disease and accurate estimation of tumor size in women with newly diagnosed ILC regardless of breast density status [127,128,129,130,131]. Studies have shown the weak radiologic-pathologic correlation on the identification and tumor size of ILC [63,132], especially in ILCs with a *CDH1* truncating mutation or null/focal expression of E-cadherin [21]. For example, a recent study by Cserni et al. [63] showed that of the 106 ILCs that the pathologists felt to be mass forming on the basis of gross and microscopic examinations, 81 (76%) were classified as mass forming, 4 (3.8%) were occult and 21 (19.8%) were architectural distortion or increase in density by radiologists. Of the 87 mass-forming mammographic abnormalities, 6 (7%) were non-mass findings on the histological examination. Four of 9 (44%) mammographically occult lesions were felt to be mass-forming on pathologic evaluation. In addition, there was no obvious correlation between histological variants of ILCs and mammographic appearance [63]. Another study of 111 patients with ILC found the correlation coefficient between pathology and different breast imaging modalities were 0.17 for mammogram, 0.37 for ultrasound and 0.58 for MRI. The actual tumor size, measured in surgical pathology specimens was underestimated by 1 cm in 27.1% of mammogram, in 50% of ultrasound, and in 13.3% of MRI [132].

Among the various imaging modalities, ultrasound is the primary method of choice worldwide to evaluate the axilla in women with newly diagnosed breast cancer. In general, the diagnostic accuracy of breast ultrasound for depicting nonpalpable metastatic lymph nodes is reported as 26–76% for sensitivity and 88–98% for specificity when morphologic characteristics are used [133,134]; however, the diagnostic accuracy is significantly reduced in patients with ILCs. In a study by Schumacher et al., the sensitivity of mammography, ultrasound, and MRI were 7%, 26%, and 7% in diagnosing axillary lymph node metastasis, with 38% of patients having false positive results for axillary lymph node metastasis [135].

Patients with ILCs are more commonly treated with mastectomy (22–52% vs. 14–46%) and are more often to have positive resection margins, especially in breast-conserving surgery specimens, when compared to ductal carcinomas (30.2% vs. 19.6%) [136]. Due to a higher incidence of diffuse spreading without significant stromal reaction and multifocality in ILCs that are challenging to detect both on imaging and intraoperative assessment, a second surgery for margin re-excision or mastectomy may be needed in up to 65% of ILCs after original breast-conserving surgery [137,138].

Since ILCs usually have low proliferation rates and high ER expression, they are less responsive than IDC to neoadjuvant chemotherapy, with low pathological complete response (pCR) rates [139]. A meta-analysis of 40 studies including 87,303 breast cancer (7596 ILCs and 79,708 IDCs) patients demonstrated when compared to their ductal counterpart, patients with ILC were far less likely to achieve pCR in the breast (7.4% vs. 22.1%, *p* < 0.00001) or axilla (13.4% vs. 23.6%, *p* < 0.00001), receive less breast-conserving surgery (33.3% vs. 45.7%, *p* < 0.00001) and more likely to have positive margins (36% vs. 13.5%, *p* < 0.00001) [140]. Compared to classic ILCs, HER2 positive and triple negative non-classic ILCs showed a greater response to neoadjuvant chemotherapy (mean residual cancer burden score (RCB): 2.46 vs. 3.41, *p* = 0.037) and higher pCR rates (15% vs. 0% *p* = 0.017), while worse overall 5-year disease-free survival [141]. pCR has been generally considered a promising prognostic factor for breast cancers after neoadjuvant chemotherapy; however, studies have found that lower pCR rates in ILCs do not necessarily lead to significantly worse outcomes when compared to ductal carcinomas [139].

With the growing knowledge of the unique tumor biology of ILCs and the distinct clinicopathological features of ILCs, clinical trials especially focusing on ILCs are gaining momentum. As listed in clinicaltrial.gov, as of 12/2024, there are six ongoing clinical trials specifically for ILC, including one trial to improve the detection of metastatic ILCs by imaging, two for biomarker testing, two for neoadjuvant treatment and one for adjuvant treatment (Table 1). Due to the characteristic loss of E-cadherin expression in lobular cancers, several preclinical studies have utilized models of *CDH1*-defective breast cancer to explore whether new targets could be specifically applied to ILC [142]. For example, the proto-oncogene *ROS1* encodes a receptor tyrosine kinase (ROS1), and a preclinical study of *CDH1* synthetic lethality interactions has identified ROS1 as a potential target in breast tumor cells with CRISPR/Cas9-engineered *CDH1* mutations [126]. The ongoing ROSALINE trial (NCT04551495) is investigating the role of potent ROS1 inhibitor Entrectinib in combination with letrozole or goserelin in the neoadjuvant setting for ILC patients [143].

## 6. Artificial Intelligence in the Detection and Diagnosis of Invasive Lobular Carcinoma

Commercial computer-aided detection (CAD) systems for screening mammography and breast ultrasound have been used to assist radiologists in the interpretation and alleviate the time- and labor-intensive manual analysis [144]. However, the potential of CAD technology in diagnosing ILCs has been rarely studied. Arce et al. retrospectively evaluated the ability of an artificial intelligence (AI) aided CAD system on 153 biopsy-proven ILCs using digital mammography and found the detection of ILC by AI CAD mammography had a sensitivity of 80%, with the highest sensitivity for detecting calcifications (100%), masses with spiculated margins (86%) and masses with irregular shape (82%) [145]. This study also revealed that 88% of mammograms had at least one false positive mark with an average number of 3.9 false positive marks per mammogram, which may confound the ability to determine the overall accuracy as well as the potential use of AI CAD in real-world practice [145]. On the contrary, a study by Raafat et al. showed AI-aided mammography performed well in detecting ILC with higher sensitivity (96.6%), and AI was more sensitive to detecting cancers with suspicious mass, suspicious calcifications, and asymmetry/distortion than mammography [146]. A recent retrospective study tested an AI decision support system on 83 biopsy-prove ILCs, and the results showed that the AI system interpreted 100% of ILCs as suspicious or probably malignant, including one case with benign features, with 100% sensitivity and 0% false-negative rate. The findings from this study suggested AI systems may evolve to detect areas of ultrasound interest for radiologist review, which would decrease interval cancers in the ILC setting [147].

Similarly, the adoption of AI-based tools in pathology, especially whole-slide imaging (WSI) has expanded significantly in recent years, with the goal of improving diagnostic accuracy and efficiency, as well as reducing tedious workloads. Currently, efforts in breast pathology have been focusing on breast cancer diagnosis and the detection of lymph node metastasis, quantitative analysis of ER, PR, HER2, Ki-67, TILs, mitotic count, and the evaluation of treatment response to neoadjuvant therapy (see review in [148]). A recent study by Challa et al. compared time consumed on reviewing IHC slides and AI-annotated images, and found that the average time consumed per AI-annotated slide was significantly less than the time consumed per IHC slide (0.6 min vs. 1.0 min; *p* = 0.0377) [149]. The same study also tested the performance of the AI algorithm on the detection of lymph node metastasis, including 21 ILC and post-neoadjuvant chemotherapy cases. AI algorithm detected all 81 metastases, including 5 cases of isolated tumor cells with a sensitivity of 100%, specificity of 78.5%, positive predictive value of 68.1%, and negative predictive value of 100% [149]. In a large, multisite validation study of AI algorithms for breast cancer detection on biopsy specimens, the AI algorithm achieved very high accuracy in differentiating subtypes of invasive carcinomas with an AUC of 0.97 between IDC and ILC and accurately diagnosed two challenging ILC cases, one with a diffuse growth pattern and the other one with associated granulomatous mastitis [150]. Interestingly, a recent study applied a *CDH1* biallelic mutations-based AI model to WSI histologic images for ILC diagnosis. The model diagnosed ILC precisely and accurately with an accuracy of 0.95 and 0.89 in their internal and external validation cohorts, respectively [151]. For the AI-based diagnosis of ILCs, rare false positive (FP) and false negative (FN) cases were noted, and the rates for FP and FN were 0.28% (3/1057) and 3.8% (40/1057) in the internal development cohort and were 0% and 5.4% (22/405) in the validation cohort. Most of the FN cases were ILC variants/mixed ductal–lobular cases (95%, 38/40 in the development cohort and 77%, 17/22 in the validation cohort) [151]. In addition, the AI model also accurately predicted *CDH1* biallelic mutations, with an accuracy of 0.95. These promising results indicate that the AI-based model can robustly classify ILCs and uncover *CDH1* inactivating mechanisms, paving the way for the development of diagnostic AI models applied to pathology that facilitate accurate cancer diagnosis and biologic discoveries.

## 7. Conclusions

In recent years, ILC has garnered increasing attention due to unique molecular alterations and resulted in distinct biological, morphological, and clinical characteristics. The application of IHCs for E-cadherin and/or associated cadherin–catenin complex, including p120-catenin and beta-catenin, especially in morphologically equivocal cases, significantly improves the diagnostic accuracy and interobserver reproducibility, as well as promotes the identification of new morphologic variants. Correlation with tumor morphology and close examination of controls and staining patterns of these IHC markers should be considered for appropriate tumor classification. ILCs exhibit unique tumor biology, and this presents considerable challenges in clinical management. Recent advances in translational and clinical research have enhanced our understanding of ILC and have spurred the development of new clinical trials specifically targeting these cancers. Additional studies are still largely needed to improve the overall outcome of ILC patients. Based on a recent worldwide survey of patients, advocates, physicians, and laboratory-based researchers, the priority research questions on ILC include: (1) refining treatment guidelines (from physicians); (2) developing large genomic data sets and ILC models (from laboratory-based researcher); (3) improvement of ILC screening and early detection, and identification of better imaging tools (from patients/advocates); and (4) improvement of understanding of endocrine resistance in ILC and identifying novel drugs that can be tested in clinical trials (from both researchers and clinicians) [152]. More specifically, from the authors’ view, collaboration in research efforts on ILC, in general, should include, but not limited to: (1) to enhance the detection of ILCs radiologically; (2) to improve the pathologically diagnostic accuracy and reproducibility of ILC; (3) to elucidate the tumor biology of ILC that is associated with late recurrence, tumor multifocality, and unusual metastatic sites; (4) to better understand the genomic landscape of ILCs, especially in patients with treatment resistance; (5) to further establish the validity of commonly-used genomic assays in ILC patients; and (6) to increase the development of clinical trials specifically designed for ILC and treatment algorithms that is more specifically address the unique tumorigenesis of ILC.

## Figures and Tables

**Table 1:** Current ongoing clinical trials specifically for invasive lobular carcinoma of the breast.

Trial	Study Title	Country	Phase	Arm	Key Inclusion Criteria	Study Summary	Start Date	Status (as of 31 December 2024)
NCT04252859	18F-Fluoroestradiol-PET/CT Imaging of ILC	U.S.	II	Single arm	Biopsy-proven primary or metastatic ILC within the past 12 weeks	To investigate the diagnostic validity of FES PET/CT imaging for ILC	11 December 2020	Recruiting
NCT06067503	Biomarkers to Detect Endocrine Therapy Resistance	U.S.	II	Single arm	ER/PR+, /HER2- metastatic ILC	To identify possible biomarkers of response to ET in ER+ metastatic ILC. Baseline levels and dynamic changes in estrogen signaling, measured by FFNP-PET/CT and liquid biopsy, will be correlated with clinical response to ET and progression-free survival	30 April 2024	Recruiting
NCT04551495	Neoadjuvant Study of Targeting ROS1 in Combination with ET in ILC of the Breast (ROSALINE)	Belgium France	II	Single arm	ER+/HER2-ILC, stage 1–3	To evaluate the efficacy of 4 months treatment of a combination of Entrectinib and ET in the neoadjuvant setting of ILC. Subjects’ response to therapy will be evaluated at baseline, two and four cycles of treatment by breast MRI, respectively	14 January 2021	Active, not recruiting
NCT05919108	Neoadjuvant Neratinib in Stage I–III HER2-Mutated Lobular Breast Cancers	U.S.	II	Two arms	Biopsy-proven HR+ ILC with documented activating HER2 mutation, stage I–III	To determine the efficacy of neoadjuvant neratinib in combination with ET in patients with HER2-mutated ILC	30 September 2024	Recruiting
NCT06408168	Phase II Study of REPotrectinib with or without Fulvestrant in HR+/HER2- Metastatic ILC	U.S.	II	Two arms	ER+/HER2- metastatic ILC patients who received a prior ET in combination with CDK4/6i	To evaluate if the combination of repotrectinib and fulvestrant can control the disease in patients with metastatic ILC	8 August 2024	Recruiting
NCT06666439	Longitudinal Tumor Burden Quantification Using Circulating Tumor DNA in Metastatic ILC	U.S.	II	Single arm	Pathologically-proven ER+/HER2- ILC with radiologic or clinical evidence of metastatic disease, treated with first-line ET	To characterize dynamic changes in circulating tumor DNA (ctDNA) during ET in metastatic ILC. Change in ctDNA is measured at baseline, 4 weeks, 8 weeks, and 12 weeks after ET treatment, respectively	12 December 2024	Recruiting

Abbreviations: CDK4/6; cyclin-dependent kinase 4 and 6; ER: estrogen receptor; ET: endocrine therapy; HER2: human epidermal growth factor receptor 2; ILC: invasive lobular carcinoma; MRI: magnetic resonance imaging; PET-CT: positron emission tomography/computed tomography; PR: progesterone receptor.

## References

[1] WHO Classification of Tumours Editorial Board (2019). Breast Tumours.

[2] Rosa M., Mohammadi A., Masood S. (2009). Lobular carcinoma of the breast with extracellular mucin: New variant of mucin-producing carcinomas?. Pathol. Int..

[3] Rakha E.A., Abbas A., Sheeran R. (2016). Invasive Lobular Carcinoma Mimicking Papillary Carcinoma: A Report of Three Cases. Pathobiol. J. Immunopathol. Mol. Cell Biol..

[4] Christgen M., Bartels S., van Luttikhuizen J.L., Schieck M., Pertschy S., Kundu S., Lehmann U., Sander B., Pelz E., Länger F. (2017). Subclonal analysis in a lobular breast cancer with classical and solid growth pattern mimicking a solid-papillary carcinoma. J. Pathol. Clin. Res..

[5] Dabbs D.J., Bhargava R., Chivukula M. (2007). Lobular versus ductal breast neoplasms: The diagnostic utility of p120 catenin. Am. J. Surg. Pathol..

[6] Cserni G. (1999). Reproducibility of a diagnosis of invasive lobular carcinoma. J. Surg. Oncol..

[7] Longacre T.A., Ennis M., Quenneville L.A., Bane A.L., Bleiweiss I.J., Carter B.A., Catelano E., Hendrickson M.R., Hibshoosh H., Layfield L.J. (2006). Interobserver agreement and reproducibility in classification of invasive breast carcinoma: An NCI breast cancer family registry study. Mod. Pathol..

[8] Christgen M., Kandt L.D., Antonopoulos W., Bartels S., Van Bockstal M.R., Bredt M., Brito M.J., Christgen H., Colpaert C., Cserni B. (2022). Inter-observer agreement for the histological diagnosis of invasive lobular breast carcinoma. J. Pathol. Clin. Res..

[9] Metzger O., Cardoso F., Poncet C., Desmedt C., Linn S., Wesseling J., Hilbers F., Aalders K., Delorenzi M., Delaloge S. (2020). Clinical utility of MammaPrint testing in Invasive Lobular Carcinoma: Results from the MINDACT phase III trial. Eur. J. Cancer.

[10] Christgen M., Gluz O., Harbeck N., Kates R.E., Raap M., Christgen H., Clemens M., Malter W., Nuding B., Aktas B. (2020). Differential impact of prognostic parameters in hormone receptor-positive lobular breast cancer. Cancer.

[11] Corso G., Fusco N., Guerini-Rocco E., Leonardi M.C., Criscitiello C., Zagami P., Nicolò E., Mazzarol G., La Vecchia C., Pesapane F. (2024). Invasive lobular breast cancer: Focus on prevention, genetics, diagnosis, and treatment. Semin. Oncol..

[12] Moll R., Mitze M., Frixen U.H., Birchmeier W. (1993). Differential loss of E-cadherin expression in infiltrating ductal and lobular breast carcinomas. Am. J. Pathol..

[13] Berx G., Cleton-Jansen A.M., Strumane K., de Leeuw W.J., Nollet F., van Roy F., Cornelisse C. (1996). E-cadherin is inactivated in a majority of invasive human lobular breast cancers by truncation mutations throughout its extracellular domain. Oncogene.

[14] Berx G., Cleton-Jansen A.M., Nollet F., de Leeuw W.J., van de Vijver M., Cornelisse C., van Roy F. (1995). E-cadherin is a tumour/invasion suppressor gene mutated in human lobular breast cancers. EMBO J..

[15] Cancer Genome Atlas Network (2012). Comprehensive molecular portraits of human breast tumours. Nature.

[16] Ciriello G., Gatza M.L., Beck A.H., Wilkerson M.D., Rhie S.K., Pastore A., Zhang H., McLellan M., Yau C., Kandoth C. (2015). Comprehensive Molecular Portraits of Invasive Lobular Breast Cancer. Cell.

[17] González-Martínez S., Kajabova V.H., Pérez-Mies B., Carretero-Barrio I., Caniego-Casas T., Sarrió D., Moreno-Bueno G., Gión M., Perez-García J., Cortés J. (2024). CDH1 methylation analysis in invasive lobular breast carcinomas with and without gene mutation. Virchows Arch. Int. J. Pathol..

[18] Morrogh M., Andrade V.P., Giri D., Sakr R.A., Paik W., Qin L.X., Arroyo C.D., Brogi E., Morrow M., King T.A. (2012). Cadherin-catenin complex dissociation in lobular neoplasia of the breast. Breast Cancer Res. Treat..

[19] Corso G., Figueiredo J., De Angelis S.P., Corso F., Girardi A., Pereira J., Seruca R., Bonanni B., Carneiro P., Pravettoni G. (2020). E-cadherin deregulation in breast cancer. J. Cell Mol. Med..

[20] Grabenstetter A., Mohanty A.S., Rana S., Zehir A., Brannon A.R., D’Alfonso T.M., DeLair D.F., Tan L.K., Ross D.S. (2020). E-cadherin immunohistochemical expression in invasive lobular carcinoma of the breast: Correlation with morphology and CDH1 somatic alterations. Hum. Pathol..

[21] Djerroudi L., Bendali A., Fuhrmann L., Benoist C., Pierron G., Masliah-Planchon J., Kieffer Y., Carton M., Tille J.-C., Cyrta J. (2024). E-Cadherin Mutational Landscape and Outcomes in Breast Invasive Lobular Carcinoma. Mod. Pathol..

[22] Takeichi M. (1977). Functional correlation between cell adhesive properties and some cell surface proteins. J. Cell Biol..

[23] Wong S.H.M., Fang C.M., Chuah L.-H., Leong C.O., Ngai S.C. (2018). E-cadherin: Its dysregulation in carcinogenesis and clinical implications. Crit. Rev. Oncol. Hematol..

[24] Qian X., Karpova T., Sheppard A.M., McNally J., Lowy D.R. (2004). E-cadherin-mediated adhesion inhibits ligand-dependent activation of diverse receptor tyrosine kinases. EMBO J..

[25] Kim N.-G., Koh E., Chen X., Gumbiner B.M. (2011). E-cadherin mediates contact inhibition of proliferation through Hippo signaling-pathway components. Proc. Natl. Acad. Sci. USA.

[26] Uchida S., Sugino T. (2024). Insights into E-Cadherin Impairment in CDH1-Unaltered Invasive Lobular Carcinoma: A Comprehensive Bioinformatic Study. Int. J. Mol. Sci..

[27] De Leeuw W.J., Berx G., Vos C.B., Peterse J.L., Van de Vijver M.J., Litvinov S., Van Roy F., Cornelisse C.J., Cleton-Jansen A.M. (1997). Simultaneous loss of E-cadherin and catenins in invasive lobular breast cancer and lobular carcinoma in situ. J. Pathol..

[28] Dopeso H., Gazzo A.M., Derakhshan F., Brown D.N., Selenica P., Jalali S., Da Cruz Paula A., Marra A., da Silva E.M., Basili T. (2024). Genomic and epigenomic basis of breast invasive lobular carcinomas lacking CDH1 genetic alterations. NPJ Precis. Oncol..

[29] Desmedt C., Zoppoli G., Gundem G., Pruneri G., Larsimont D., Fornili M., Fumagalli D., Brown D., Rothé F., Vincent D. (2016). Genomic Characterization of Primary Invasive Lobular Breast Cancer. J. Clin. Oncol. Off. J. Am. Soc. Clin. Oncol..

[30] de Groot J.S., Ratze M.A., van Amersfoort M., Eisemann T., Vlug E.J., Niklaas M.T., Chin S.-F., Caldas C., van Diest P.J., Jonkers J. (2018). αE-catenin is a candidate tumor suppressor for the development of E-cadherin-expressing lobular-type breast cancer. J. Pathol..

[31] Canas-Marques R., Schnitt S.J. (2016). E-cadherin immunohistochemistry in breast pathology: Uses and pitfalls. Histopathology.

[32] Dabbs D.J., Kaplai M., Chivukula M., Kanbour A., Kanbour-Shakir A., Carter G.J. (2007). The spectrum of morphomolecular abnormalities of the E-cadherin/catenin complex in pleomorphic lobular carcinoma of the breast. Appl. Immunohistochem. Mol. Morphol. AIMM.

[33] Sarrió D., Pérez-Mies B., Hardisson D., Moreno-Bueno G., Suárez A., Cano A., Martín-Pérez J., Gamallo C., Palacios J. (2004). Cytoplasmic localization of p120ctn and E-cadherin loss characterize lobular breast carcinoma from preinvasive to metastatic lesions. Oncogene.

[34] Foote F.W., Stewart F.W. (1941). Lobular carcinoma in situ: A rare form of mammary cancer. Am. J. Pathol..

[35] Iorfida M., Maiorano E., Orvieto E., Maisonneuve P., Bottiglieri L., Rotmensz N., Montagna E., Dellapasqua S., Veronesi P., Galimberti V. (2012). Invasive lobular breast cancer: Subtypes and outcome. Breast Cancer Res. Treat..

[36] Makhlouf S., Atallah N.M., Polotto S., Lee A.H.S., Green A.R., Rakha E.A. (2024). Deciphering the Clinical Behaviour of Invasive Lobular Carcinoma of the Breast Defines an Aggressive Subtype. Cancers.

[37] Christgen M., Steinemann D., Kühnle E., Länger F., Gluz O., Harbeck N., Kreipe H. (2016). Lobular breast cancer: Clinical, molecular and morphological characteristics. Pathol. Res. Pract..

[38] Yu J., Dabbs D.J., Shuai Y., Niemeier L.A., Bhargava R. (2011). Classical-type invasive lobular carcinoma with HER2 overexpression: Clinical, histologic, and hormone receptor characteristics. Am. J. Clin. Pathol..

[39] Hood C.I., Font R.L., Zimmerman L.E. (1973). Metastatic mammary carcinoma in the eyelid with histiocytoid appearance. Cancer.

[40] Soong T.R., Dillon D.A., Rice-Stitt T.L., Wieczorek T.J., Baker G.M., Darvishian F., Collins L.C., Lester S.C., Schnitt S.J., Harrison B.T. (2022). Invasive lobular carcinoma with extracellular mucin (ILCEM): Clinicopathologic and molecular characterization of a rare entity. Mod. Pathol..

[41] Yu J., Bhargava R., Dabbs D.J. (2010). Invasive lobular carcinoma with extracellular mucin production and HER-2 overexpression: A case report and further case studies. Diagn. Pathol..

[42] Cserni G., Floris G., Koufopoulos N., Kovács A., Nonni A., Regitnig P., Stahls A., Varga Z. (2017). Invasive lobular carcinoma with extracellular mucin production-a novel pattern of lobular carcinomas of the breast. Clinico-pathological description of eight cases. Virchows Arch. Int. J. Pathol..

[43] Haltas H., Bayrak R., Yenidunya S., Kosehan D., Sen M., Akin K. (2012). Invasive lobular carcinoma with extracellular mucin as a distinct variant of lobular carcinoma: A case report. Diagn. Pathol..

[44] Bari V.B., Bholay S.U., Sane K.C. (2015). Invasive lobular carcinoma of the breast with extracellular mucin- a new rare variant. J. Clin. Diagn. Res. JCDR.

[45] Gómez Macías G.S., Pérez Saucedo J.E., Cardona Huerta S., Garza Montemayor M., Villarreal Garza C., García Hernández I. (2016). Invasive lobular carcinoma of the breast with extracellular mucin: A case report. Int. J. Surg. Case Rep..

[46] Boukhechba M., Kadiri H., El Khannoussi B. (2018). Invasive Lobular Carcinoma of the Breast with Extracellular Mucin: Case Report of a New Variant of Lobular Carcinoma of the Breast. Case Rep. Pathol..

[47] Singh K., DiazGomez B., Wang Y., Ou J., Hansen K. (2019). Invasive Lobular Carcinoma With Extracellular Mucin: Not All Mucinous Mammary Carcinomas Are Ductal!. Int. J. Surg. Pathol..

[48] Koufopoulos N., Antoniadou F., Kokkali S., Pigadioti E., Khaldi L. (2019). Invasive Lobular Carcinoma with Extracellular Mucin Production: Description of a Case and Review of the Literature. Cureus.

[49] Hort A., O’Toole S.A., Yunaev M. (2022). An unusual case of invasive lobular carcinoma with abundant extracellular mucin. Pathology.

[50] Motanagh S.A., Muller K.E. (2020). Invasive lobular carcinoma with papillary features: A newly described variant that poses a difficult histologic differential diagnosis. Breast J..

[51] Gessain G., Joyon N., Petit T., Cotteret S., Lacroix-Triki M. (2023). Uncommon invasive lobular carcinoma with papillary architecture-clinicopathologic and molecular characterization with review of the literature. Virchows Arch. Int. J. Pathol..

[52] Zheng L., Saluja K., Guo T. (2022). Invasive Lobular Carcinoma Mimicking Encapsulated Papillary Carcinoma with a Literature Review: A Rare Variant Detected Serendipitously. Int. J. Surg. Pathol..

[53] Kuba M.G., Brogi E. (2023). Update on lobular lesions of the breast. Histopathology.

[54] Li X., Lin M., Xu J., Pang Y., Chen H., Sun P., Hou G. (2021). New variant of breast-invasive lobular carcinoma with solid and encapsulated papillary carcinoma growth pattern. Breast Cancer Tokyo Jpn..

[55] Christgen M., Bartels S., van Luttikhuizen J.L., Bublitz J., Rieger L.U., Christgen H., Stark H., Sander B., Lehmann U., Steinemann D. (2020). E-cadherin to P-cadherin switching in lobular breast cancer with tubular elements. Mod. Pathol..

[56] Wheeler D.T., Tai L.H., Bratthauer G.L., Waldner D.L., Tavassoli F.A. (2004). Tubulolobular carcinoma of the breast: An analysis of 27 cases of a tumor with a hybrid morphology and immunoprofile. Am. J. Surg. Pathol..

[57] Kuroda H., Tamaru J.-I., Takeuchi I., Ohnisi K., Sakamoto G., Adachi A., Kaneko K., Itoyama S. (2006). Expression of E-cadherin, alpha-catenin, and beta-catenin in tubulolobular carcinoma of the breast. Virchows Arch. Int. J. Pathol..

[58] Esposito N.N., Chivukula M., Dabbs D.J. (2007). The ductal phenotypic expression of the E-cadherin/catenin complex in tubulolobular carcinoma of the breast: An immunohistochemical and clinicopathologic study. Mod. Pathol..

[59] Weidner N., Semple J.P. (1992). Pleomorphic variant of invasive lobular carcinoma of the breast. Hum. Pathol..

[60] Bentz J.S., Yassa N., Clayton F. (1998). Pleomorphic lobular carcinoma of the breast: Clinicopathologic features of 12 cases. Mod. Pathol..

[61] Rakha E.A., van Deurzen C.H.M., Paish E.C., Macmillan R.D., Ellis I.O., Lee A.H.S. (2013). Pleomorphic lobular carcinoma of the breast: Is it a prognostically significant pathological subtype independent of histological grade?. Mod. Pathol..

[62] Monhollen L., Morrison C., Ademuyiwa F.O., Chandrasekhar R., Khoury T. (2012). Pleomorphic lobular carcinoma: A distinctive clinical and molecular breast cancer type. Histopathology.

[63] Cserni G., Bori R., Ambrózay É., Serfőző O. (2024). Histological Patterns and Mammographic Presentation of Invasive Lobular Carcinoma Show No Obvious Associations. Cancers.

[64] Tille J.-C., Vieira A.F., Saint-Martin C., Djerroudi L., Furhmann L., Bidard F.-C., Kirova Y., Tardivon A., Reyal F., Carton M. (2020). Tumor-infiltrating lymphocytes are associated with poor prognosis in invasive lobular breast carcinoma. Mod. Pathol..

[65] Kiaer H., Andersen J.A., Rank F., Pedersen B.V. (1988). Quality control of patho-anatomical diagnosis of carcinoma of the breast. Acta Oncol. Stockh. Swed..

[66] Rasbridge S.A., Gillett C.E., Sampson S.A., Walsh F.S., Millis R.R. (1993). Epithelial (E-) and placental (P-) cadherin cell adhesion molecule expression in breast carcinoma. J. Pathol..

[67] Gamallo C., Palacios J., Suarez A., Pizarro A., Navarro P., Quintanilla M., Cano A. (1993). Correlation of E-cadherin expression with differentiation grade and histological type in breast carcinoma. Am. J. Pathol..

[68] Cserni G., Kálmán E., Udvarhelyi N., Papp E., Grote I., Bartels S., Christgen M., Kreipe H., Kulka J. (2023). Evaluation of the routine use of E-cadherin immunohistochemistry in the typing of breast carcinomas: Results of a randomized diagnostic study. Histopathology.

[69] De Schepper M., Koorman T., Richard F., Christgen M., Vincent-Salomon A., Schnitt S.J., van Diest P.J., Zels G., Mertens F., Maetens M. (2024). Integration of Pathological Criteria and Immunohistochemical Evaluation for Invasive Lobular Carcinoma Diagnosis: Recommendations From the European Lobular Breast Cancer Consortium. Mod. Pathol..

[70] De Schepper M., Vincent-Salomon A., Christgen M., Van Baelen K., Richard F., Tsuda H., Kurozumi S., Brito M.J., Cserni G., Schnitt S. (2022). Results of a worldwide survey on the currently used histopathological diagnostic criteria for invasive lobular breast cancer. Mod. Pathol..

[71] Dabbs D.J., Schnitt S.J., Geyer F.C., Weigelt B., Baehner F.L., Decker T., Eusebi V., Fox S.B., Ichihara S., Lakhani S.R. (2013). Lobular neoplasia of the breast revisited with emphasis on the role of E-cadherin immunohistochemistry. Am. J. Surg. Pathol..

[72] Dillon D.A., D’Aquila T., Reynolds A.B., Fearon E.R., Rimm D.L. (1998). The expression of p120ctn protein in breast cancer is independent of alpha- and beta-catenin and E-cadherin. Am. J. Pathol..

[73] Calhoun B.C., Dabbs D.J. (2023). Lack of Standardization in the Diagnosis of Invasive Lobular Carcinoma of the Breast. Mod. Pathol..

[74] Rakha E.A., Abd El Rehim D., Pinder S.E., Lewis S.A., Ellis I.O. (2005). E-cadherin expression in invasive non-lobular carcinoma of the breast and its prognostic significance. Histopathology.

[75] Alsaleem M., Toss M.S., Joseph C., Aleskandarany M., Kurozumi S., Alshankyty I., Ogden A., Rida P.C.G., Ellis I.O., Aneja R. (2019). The molecular mechanisms underlying reduced E-cadherin expression in invasive ductal carcinoma of the breast: High throughput analysis of large cohorts. Mod. Pathol..

[76] Pestalozzi B.C., Zahrieh D., Mallon E., Gusterson B.A., Price K.N., Gelber R.D., Holmberg S.B., Lindtner J., Snyder R., Thürlimann B. (2008). Distinct clinical and prognostic features of infiltrating lobular carcinoma of the breast: Combined results of 15 International Breast Cancer Study Group clinical trials. J. Clin. Oncol..

[77] Corona S.P., Bortul M., Scomersi S., Bigal C., Bottin C., Zanconati F., Fox S.B., Giudici F., Generali D. (2020). Management of the axilla in breast cancer: Outcome analysis in a series of ductal versus lobular invasive cancers. Breast Cancer Res. Treat..

[78] Oesterreich S., Nasrazadani A., Zou J., Carleton N., Onger T., Wright M.D., Li Y., Demanelis K., Ramaswamy B., Tseng G. (2022). Clinicopathological Features and Outcomes Comparing Patients With Invasive Ductal and Lobular Breast Cancer. J. Natl. Cancer Inst..

[79] Biglia N., Maggiorotto F., Liberale V., Bounous V.E., Sgro L.G., Pecchio S., D’Alonzo M., Ponzone R. (2013). Clinical-pathologic features, long term-outcome and surgical treatment in a large series of patients with invasive lobular carcinoma (ILC) and invasive ductal carcinoma (IDC). Eur. J. Surg. Oncol..

[80] Mejdahl M.K., Wohlfahrt J., Holm M., Knoop A.S., Tjønneland A., Melbye M., Kroman N., Balslev E. (2020). Synchronous bilateral breast cancer: A nationwide study on histopathology and etiology. Breast Cancer Res. Treat..

[81] Ashikari R., Huvos A.G., Urban J.A., Robbins G.F. (1973). Infiltrating lobular carcinoma of the breast. Cancer.

[82] Arpino G., Bardou V.J., Clark G.M., Elledge R.M. (2004). Infiltrating lobular carcinoma of the breast: Tumor characteristics and clinical outcome. Breast Cancer Res. BCR.

[83] Verboven G., Lodewijkx I., Van den Bosch L., Huizing M., Van Goethem M., Broeckx G., Tjalma W.A. (2024). Literature review on the bilateral occurrence of invasive lobular breast cancer. Eur. J. Obstet. Gynecol. Reprod. Biol..

[84] Duraker N., Hot S., Akan A., Nayır P.Ö. (2020). A Comparison of the Clinicopathological Features, Metastasis Sites and Survival Outcomes of Invasive Lobular, Invasive Ductal and Mixed Invasive Ductal and Lobular Breast Carcinoma. Eur. J. Breast Health.

[85] DiPiro P.J., Tirumani S.H., Cruz G.P., Ramaiya N.H., Lester S.C., Shinagare A.B. (2019). Lobular breast cancer: Patterns of intraabdominal metastatic spread on imaging and prognostic significance. Abdom. Radiol..

[86] Mouabbi J.A., Hassan A., Lim B., Hortobagyi G.N., Tripathy D., Layman R.M. (2022). Invasive lobular carcinoma: An understudied emergent subtype of breast cancer. Breast Cancer Res. Treat..

[87] McCart Reed A.E., Kalinowski L., Simpson P.T., Lakhani S.R. (2021). Invasive lobular carcinoma of the breast: The increasing importance of this special subtype. Breast Cancer Res. BCR.

[88] Shousha S., Backhous C.M., Alaghband-Zadeh J., Burn I. (1986). Alveolar variant of invasive lobular carcinoma of the breast. A tumor rich in estrogen receptors. Am. J. Clin. Pathol..

[89] Mehdi M., Kong A.L., Frebault J., Huang S., Huang C.-C., Cortina C.S. (2021). Prognostic Outcomes of Signet Ring Cell Carcinoma of the Breast. J. Surg. Res..

[90] Eltorky M., Hall J.C., Osborne P.T., el Zeky F. (1994). Signet-ring cell variant of invasive lobular carcinoma of the breast. A clinicopathologic study of 11 cases. Arch. Pathol. Lab. Med..

[91] Coty-Fattal Z., Minhas S., Butcher M., Agarwal I., LaBoy C., Blanco L., Novo J. (2024). Clinicopathologic and Immunophenotypic Classification of Invasive Lobular Carcinoma with Histiocytoid Features. Int. J. Surg. Pathol..

[92] Kontogiannis A., Karaviti E., Karaviti D., Lanitis S., Gomatou G., Syrigos N.K., Kotteas E. (2024). Mutations Matter: Unravelling the Genetic Blueprint of Invasive Lobular Carcinoma for Progression Insights and Treatment Strategies. Cancers.

[93] Zhu S., Ward B.M., Yu J., Matthew-Onabanjo A.N., Janusis J., Hsieh C.-C., Tomaszewicz K., Hutchinson L., Zhu L.J., Kandil D. (2018). IRS2 mutations linked to invasion in pleomorphic invasive lobular carcinoma. JCI Insight.

[94] Zhang H., Moisini I., Ajabnoor R.M., Turner B.M., D’aguiar M., Cai X., Gao S., Yang Q., Wang X., Schiffhauer L. (2020). Frequency, Clinicopathologic Characteristics, and Follow-up of HER2-Positive Nonpleomorphic Invasive Lobular Carcinoma of the Breast. Am. J. Clin. Pathol..

[95] He L., Araj E., Peng Y. (2021). HER2 Positive and HER2 Negative Classical Type Invasive Lobular Carcinomas: Comparison of Clinicopathologic Features. Curr. Oncol. Tor. Ont.

[96] Forster-Sack M., Zoche M., Pestalozzi B., Witzel I., Schwarz E.I., Herzig J.J., Fansa H., Tausch C., Ross J., Moch H. (2024). ERBB2-amplified lobular breast carcinoma exhibits concomitant CDK12 co-amplification associated with poor prognostic features. J. Pathol. Clin. Res..

[97] Zhang Y., Luo X., Chen M., Yang L., Lei T., Pu T., Wei B., Bu H., Zhang Z. (2022). Biomarker profile of invasive lobular carcinoma: Pleomorphic versus classic subtypes, clinicopathological characteristics and prognosis analyses. Breast Cancer Res. Treat..

[98] Lien H.-C., Chen Y.-L., Juang Y.-L., Jeng Y.-M. (2015). Frequent alterations of HER2 through mutation, amplification, or overexpression in pleomorphic lobular carcinoma of the breast. Breast Cancer Res. Treat..

[99] Haque W., Arms A., Verma V., Hatch S., Brian Butler E., Teh B.S. (2019). Outcomes of pleomorphic lobular carcinoma versus invasive lobular carcinoma. Breast Edinb. Scotl..

[100] Jongen L., Floris G., Boeckx B., Smeets D., Lambrechts D., Vander Borght S., Laenen A., Mann G., Cutler R.E., Lalani A.S. (2019). Identification, clinical-pathological characteristics and treatment outcomes of patients with metastatic breast cancer and somatic human epidermal growth factor receptor 2 (ERBB2) mutations. Breast Cancer Res. Treat..

[101] Bose R., Kavuri S.M., Searleman A.C., Shen W., Shen D., Koboldt D.C., Monsey J., Goel N., Aronson A.B., Li S. (2013). Activating HER2 mutations in HER2 gene amplification negative breast cancer. Cancer Discov..

[102] Deniziaut G., Tille J.C., Bidard F.-C., Vacher S., Schnitzler A., Chemlali W., Trémoulet L., Fuhrmann L., Cottu P., Rouzier R. (2016). ERBB2 mutations associated with solid variant of high-grade invasive lobular breast carcinomas. Oncotarget.

[103] Shah S.P., Morin R.D., Khattra J., Prentice L., Pugh T., Burleigh A., Delaney A., Gelmon K., Guliany R., Senz J. (2009). Mutational evolution in a lobular breast tumour profiled at single nucleotide resolution. Nature.

[104] Ross J.S., Wang K., Sheehan C.E., Boguniewicz A.B., Otto G., Downing S.R., Sun J., He J., Curran J.A., Ali S. (2013). Relapsed classic E-cadherin (CDH1)-mutated invasive lobular breast cancer shows a high frequency of HER2 (ERBB2) gene mutations. Clin. Cancer Res..

[105] Joshi U., Budhathoki P., Gaire S., Yadav S.K., Shah A., Adhikari A., Choong G., Couzi R., Giridhar K.V., Leon-Ferre R.A. (2023). Clinical outcomes and prognostic factors in triple-negative invasive lobular carcinoma of the breast. Breast Cancer Res. Treat..

[106] Conforti F., Pala L., Pagan E., Rocco E.G., Bagnardi V., Montagna E., Peruzzotti G., De Pas T., Fumagalli C., Pileggi S. (2021). Biological and clinical features of triple negative Invasive Lobular Carcinomas of the breast. Clinical outcome and actionable molecular alterations. Breast Edinb. Scotl..

[107] Trillo P., Sandoval J., Trapani D., Nicolò E., Zagami P., Giugliano F., Tarantino P., Vivanet G., Ascione L., Friedlaender A. (2023). Evolution of biological features of invasive lobular breast cancer: Comparison between primary tumour and metastases. Eur. J. Cancer Oxf. Engl. 1990.

[108] Modi S., Jacot W., Yamashita T., Sohn J., Vidal M., Tokunaga E., Tsurutani J., Ueno N.T., Prat A., Chae Y.S. (2022). Trastuzumab Deruxtecan in Previously Treated HER2-Low Advanced Breast Cancer. N. Engl. J. Med..

[109] Douganiotis G., Kontovinis L., Markopoulou E., Ainali A., Zarampoukas T., Natsiopoulos I., Papazisis K. (2022). Prognostic Significance of Low HER2 Expression in Patients With Early Hormone Receptor Positive Breast Cancer. Cancer Diagn. Progn..

[110] Mutai R., Barkan T., Moore A., Sarfaty M., Shochat T., Yerushalmi R., Stemmer S.M., Goldvaser H. (2021). Prognostic impact of HER2-low expression in hormone receptor positive early breast cancer. Breast Edinb. Scotl..

[111] Rothschild H.T., Clelland E., Patterson A., Molina-Vega J., Kaur M., Symmans W.F., Schwartz C.J., Chien A.J., Mukhtar R.A. (2023). HER-2 low status in early-stage invasive lobular carcinoma of the breast: Associated factors and outcomes in an institutional series. Breast Cancer Res. Treat..

[112] Djerroudi L., El Sabeh-Ayoun A., Benoist C., Pierron G., Masliah-Planchon J., Fuhrmann L., Kieffer Y., Carton M., Ramtohul T., Callens C. (2024). Molecular and Clinical Portrait of HER2-low Invasive Lobular Carcinomas. Mod. Pathol..

[113] Göker M., Denys H., van de Vijver K., Braems G. (2022). Genomic assays for lobular breast carcinoma. J. Clin. Transl. Res..

[114] Felts J.L., Zhu J., Han B., Smith S.J., Truica C.I. (2017). An Analysis of Oncotype DX Recurrence Scores and Clinicopathologic Characteristics in Invasive Lobular Breast Cancer. Breast J..

[115] Barni S., Fabi A., Petrelli F. (2023). Lobular Carcinoma of the Breast and Utility of Oncotype Dx®: A Retrospective Decision Impact Analysis. Clin. Breast Cancer.

[116] Abel M.K., Shui A.M., Melisko M., Chien A.J., Yoshida E.J., Lancaster E.M., Van ’t Veer L., Esserman L.J., Mukhtar R.A. (2021). The incidence of discordant clinical and genomic risk in patients with invasive lobular or ductal carcinoma of the breast: A National Cancer Database Study. NPJ Breast Cancer.

[117] Desmedt C., Salgado R., Fornili M., Pruneri G., Van den Eynden G., Zoppoli G., Rothé F., Buisseret L., Garaud S., Willard-Gallo K. (2018). Immune Infiltration in Invasive Lobular Breast Cancer. J. Natl. Cancer Inst..

[118] Göker M., Deblaere S., Denys H., Vergauwen G., Naert E., Veldeman L., Monten C., Van den Broecke R., Van Dorpe J., Braems G. (2023). Tumor-Infiltrating Lymphocytes and PD-L1 Expression in Pleomorphic Lobular Breast Carcinoma. Cancers.

[119] Voorwerk L., Isaeva O.I., Horlings H.M., Balduzzi S., Chelushkin M., Bakker N.A.M., Champanhet E., Garner H., Sikorska K., Loo C.E. (2023). PD-L1 blockade in combination with carboplatin as immune induction in metastatic lobular breast cancer: The GELATO trial. Nat. Cancer.

[120] Van Baelen K., Geukens T., Maetens M., Tjan-Heijnen V., Lord C.J., Linn S., Bidard F.-C., Richard F., Yang W.W., Steele R.E. (2022). Current and future diagnostic and treatment strategies for patients with invasive lobular breast cancer. Ann. Oncol. Off. J. Eur. Soc. Med. Oncol..

[121] Coffey K., Berg W.A., Dodelzon K., Jochelson M.S., Mullen L.A., Parikh J.R., Hutcheson L., Grimm L.J. (2024). Breast Radiologists’ Perceptions on the Detection and Management of Invasive Lobular Carcinoma: Most Agree Imaging Beyond Mammography Is Warranted. J. Breast Imaging.

[122] Weaver O., Yang W. (2020). Imaging of Breast Cancers With Predilection for Nonmass Pattern of Growth: Invasive Lobular Carcinoma and DCIS-Does Imaging Capture It All?. AJR Am. J. Roentgenol..

[123] Berg W.A., Gutierrez L., NessAiver M.S., Carter W.B., Bhargavan M., Lewis R.S., Ioffe O.B. (2004). Diagnostic accuracy of mammography, clinical examination, US, and MR imaging in preoperative assessment of breast cancer. Radiology.

[124] Butler R.S., Venta L.A., Wiley E.L., Ellis R.L., Dempsey P.J., Rubin E. (1999). Sonographic evaluation of infiltrating lobular carcinoma. AJR Am. J. Roentgenol..

[125] Selinko V.L., Middleton L.P., Dempsey P.J. (2004). Role of sonography in diagnosing and staging invasive lobular carcinoma. J. Clin. Ultrasound JCU.

[126] Paramagul C.P., Helvie M.A., Adler D.D. (1995). Invasive lobular carcinoma: Sonographic appearance and role of sonography in improving diagnostic sensitivity. Radiology.

[127] Pereslucha A.M., Wenger D.M., Morris M.F., Aydi Z.B. (2023). Invasive Lobular Carcinoma: A Review of Imaging Modalities with Special Focus on Pathology Concordance. Healthcare.

[128] Muradali D., Fletcher G.G., Cordeiro E., Fienberg S., George R., Kulkarni S., Seely J.M., Shaheen R., Eisen A. (2023). Preoperative Breast Magnetic Resonance Imaging: An Ontario Health (Cancer Care Ontario) Clinical Practice Guideline. Curr. Oncol. Tor. Ont.

[129] Aroney S., Lloyd T., Birch S., Godwin B., Walters K., Khoo J., Geere S., Shen L., Vujovic P., Bennett I. (2024). Preoperative breast MR imaging influences surgical management in patients with invasive lobular carcinoma. J. Med. Imaging Radiat. Oncol..

[130] Melvin Z., Lim D., Jacques A., Falkner N.M., Lo G. (2024). Is staging breast magnetic resonance imaging for invasive lobular carcinoma worthwhile?. ANZ J. Surg..

[131] Willen L.P.A., Spiekerman van Weezelenburg M.A., Bruijsten A.A., Broos P.P.H.L., van Haaren E.R.M., Janssen A., Vissers Y.L.J., van Bastelaar J. (2024). The Role of Magnetic Resonance Imaging in the Preoperative Staging and Treatment of Invasive Lobular Carcinoma. Clin. Breast Cancer.

[132] Ozcan L.C., Donovan C.A., Srour M., Chung A., Mirocha J., Frankel S.D., Hakim P., Giuliano A.E., Amersi F. (2023). Invasive Lobular Carcinoma-Correlation Between Imaging and Final Pathology: Is MRI Better?. Am. Surg..

[133] Chang J.M., Leung J.W.T., Moy L., Ha S.M., Moon W.K. (2020). Axillary Nodal Evaluation in Breast Cancer: State of the Art. Radiology.

[134] Choi H.Y., Park M., Seo M., Song E., Shin S.Y., Sohn Y.-M. (2017). Preoperative Axillary Lymph Node Evaluation in Breast Cancer: Current Issues and Literature Review. Ultrasound Q..

[135] Schumacher K., Inciardi M., O’Neil M., Wagner J.L., Shah I., Amin A.L., Balanoff C.R., Larson K.E. (2021). Is axillary imaging for invasive lobular carcinoma accurate in determining clinical node staging?. Breast Cancer Res. Treat..

[136] O’Connor D.J., Davey M.G., McFeetors C., McLaughlin R.P., Sweeney K.J., Barry M.K., Malone C.M., Wahab S.A.E., Lowery A.J., Kerin M.J. (2024). Evaluating Surgical Outcomes Between Estrogen Receptor Positive Invasive Lobular and Invasive Ductal Carcinoma of the Breast-A Propensity Matched Analysis. Clin. Breast Cancer.

[137] Luveta J., Parks R.M., Heery D.M., Cheung K.-L., Johnston S.J. (2020). Invasive Lobular Breast Cancer as a Distinct Disease: Implications for Therapeutic Strategy. Oncol. Ther..

[138] Braunstein L.Z., Brock J.E., Chen Y.-H., Truong L., Russo A.L., Arvold N.D., Harris J.R. (2015). Invasive lobular carcinoma of the breast: Local recurrence after breast-conserving therapy by subtype approximation and surgical margin. Breast Cancer Res. Treat..

[139] Cristofanilli M., Gonzalez-Angulo A., Sneige N., Kau S.-W., Broglio K., Theriault R.L., Valero V., Buzdar A.U., Kuerer H., Buchholz T.A. (2005). Invasive lobular carcinoma classic type: Response to primary chemotherapy and survival outcomes. J. Clin. Oncol..

[140] O’Connor D.J., Davey M.G., Barkley L.R., Kerin M.J. (2022). Differences in sensitivity to neoadjuvant chemotherapy among invasive lobular and ductal carcinoma of the breast and implications on surgery—A systematic review and meta-analysis. Breast Edinb. Scotl..

[141] Quirke N.P., Cullinane C., Turk M.A., Shafique N., Evoy D., Geraghty J., McCartan D., Quinn C., Walshe J.M., McDermott E. (2024). Invasive lobular carcinoma of the breast; clinicopathologic profile and response to neoadjuvant chemotherapy over a 15-year period. Breast Edinb. Scotl..

[142] Bajrami I., Marlow R., van de Ven M., Brough R., Pemberton H.N., Frankum J., Song F., Rafiq R., Konde A., Krastev D.B. (2018). E-Cadherin/ROS1 Inhibitor Synthetic Lethality in Breast Cancer. Cancer Discov..

[143] Agostinetto E., Nader-Marta G., Paesmans M., Ameye L., Veys I., Buisseret L., Neven P., Taylor D., Fontaine C., Duhoux F.P. (2022). ROSALINE: A phase II, neoadjuvant study targeting ROS1 in combination with endocrine therapy in invasive lobular carcinoma of the breast. Future Oncol. Lond. Engl..

[144] Giger M.L., Chan H.-P., Boone J. (2008). Anniversary paper: History and status of CAD and quantitative image analysis: The role of Medical Physics and AAPM. Med. Phys..

[145] Arce S., Vijay A., Yim E., Spiguel L.R., Hanna M. (2023). Evaluation of an Artificial Intelligence System for Detection of Invasive Lobular Carcinoma on Digital Mammography. Cureus.

[146] Raafat M., Mansour S., Kamal R., Ali H.W., Shibel P.E., Marey A., Taha S.N., Alkalaawy B.A. (2022). Does artificial intelligence aid in the detection of different types of breast cancer?. Egypt. J. Radiol. Nucl. Med..

[147] Amir T., Coffey K., Sevilimedu V., Fardanesh R., Mango V.L. (2023). A role for breast ultrasound Artificial Intelligence decision support in the evaluation of small invasive lobular carcinomas. Clin. Imaging.

[148] Soliman A., Li Z., Parwani A.V. (2024). Artificial intelligence’s impact on breast cancer pathology: A literature review. Diagn. Pathol..

[149] Challa B., Tahir M., Hu Y., Kellough D., Lujan G., Sun S., Parwani A.V., Li Z. (2023). Artificial Intelligence-Aided Diagnosis of Breast Cancer Lymph Node Metastasis on Histologic Slides in a Digital Workflow. Mod. Pathol..

[150] Sandbank J., Bataillon G., Nudelman A., Krasnitsky I., Mikulinsky R., Bien L., Thibault L., Albrecht Shach A., Sebag G., Clark D.P. (2022). Validation and real-world clinical application of an artificial intelligence algorithm for breast cancer detection in biopsies. NPJ Breast Cancer.

[151] Pareja F., Dopeso H., Wang Y.K., Gazzo A.M., Brown D.N., Banerjee M., Selenica P., Bernhard J.H., Derakhshan F., da Silva E.M. (2024). A Genomics-Driven Artificial Intelligence-Based Model Classifies Breast Invasive Lobular Carcinoma and Discovers CDH1 Inactivating Mechanisms. Cancer Res..

[152] Oesterreich S., Pate L., Lee A.V., Chen F., Jankowitz R.C., Mukhtar R., Metzger O., Sikora M.J., Li C.I., Sotiriou C. (2024). International survey on invasive lobular breast cancer identifies priority research questions. NPJ Breast Cancer.

